# Identifying Organic–Inorganic Interaction Sites Toward Emission Enhancement in Non-Hydrogen-Bonded Hybrid Perovskite via Pressure Engineering

**DOI:** 10.34133/research.0476

**Published:** 2024-09-16

**Authors:** Ming Cong, Dianlong Zhao, Jiayi Yang, Guanjun Xiao, Bo Zou

**Affiliations:** State Key Laboratory of Superhard Materials, College of Physics, Jilin University, Changchun 130012, China.

## Abstract

The interaction between organic and inorganic components in metal hybrid perovskites fundamentally determines the intrinsic optoelectronic performance. However, the underlying interaction sites have still remained elusive, especially for those non-hydrogen-bonded hybrid perovskites, thus largely impeding materials precise design with targeted properties. Herein, high pressure is utilized to elucidate the interaction mechanism between organic and inorganic components in the as-synthesized one-dimensional hybrid metal halide (DBU)PbBr_3_ (DBU = 1,8-diazabicyclo [5.4.0] undec-7-ene). The interaction sites are identified to be the N from DBU and the Br from inorganic framework by the indicative of enhanced Raman mode under high pressure. The change in interaction strength is indeed derived from the pressure modulation on both distance and spatial arrangement of the nearest Br and N, rather than traditional hydrogen-bonding effect. Furthermore, the enhanced interaction increased charge transfer, resulting in a cyan emission with photoluminescence quantum yields (PLQYs) of 86.6%. The enhanced cyan emission is particularly important for underwater communication due to the much less attenuation in water than at other wavelength emissions. This study provides deep insights into the underlying photophysical mechanism of non-hydrogen-bonded hybrid metal halides and is expected to impart innovative construction with superior performance.

## Introduction

Low-dimensional hybrid metal halides (LHMHs) have been highly coveted in solid-state lighting applications for their excellent photoelectric properties recently [1–7]. Strong electron–phonon coupling usually leads to broadband self-trapped exciton (STE) emission with large Stokes shift [8–13]. For most of LHMHs, their organic components do not contribute to the optical properties, but rather act as spatial isolators without markedly interacting with the inorganic components [14–16]. For a portion of the LHMHs, the organic components interact strongly with the inorganic frameworks, accordingly determining the eventual STE emission [17–20]. It has been proven that introducing conjugated organic molecules into LHMHs can enhance the charge transfer and lead to superior performance [18,21]. However, the underlying interaction mechanisms are just vaguely attributed to the charge transfer [19], and the specific interaction sites remain elusive, especially for the non-hydrogen-bonded LHMHs. This would greatly impede the precise design and synthesis of highly luminescent LHMH materials. In addition, materials that emit cyan are particularly important for underwater communication, as light in this wavelength range is much less attenuated in water than at other wavelengths. Accordingly, cyan-emitting materials are of great urgent candidate for high-quality lighting and light communication.

High pressure, as an alternative thermodynamic variable, can effectively reduce interatomic distances and modify bonding patterns of materials to achieve superior optical properties without inducing other chemical compositions [22–27]. In addition, pressure provides a potent means to address the debates under environmental conditions [28–33]. Under atmospheric pressure, the green emission of Cs_4_PbBr_6_ NCs was confirmed to originate from the CsPbBr_3_ embedded in the lattice by introducing pressure dimension [34]. Subsequently, the origin of blue light of indium-based double perovskites was clarified as self-trapped singlet exciton radiative recombination via pressure engineering [35]. Furthermore, pressure was employed to clarify the origin of the defect emission in chalcogenide quantum dots, in which the surface hole traps predominantly contributed to radiative defect emission [36]. Likewise, pressure engineering enabled the possibility to modulate the space arrangement of atomics, and thus regulating the organic and inorganic interaction to design novel LHMHs [19,37–39]. However, most of the previous studies were focused on the LHMHs with hydrogen bond existing, which limited guides for the larger number of non-hydrogen-bonded LHMHs. Accordingly, we sought to investigate the specific sites of the organic–inorganic interaction in non-hydrogen-bonded LHMHs by invoking high pressure.

Here, we successfully revealed the organic–inorganic interaction site and its influence on the optical properties in the as-prepared one-dimensional (1D) hybrid metal halide (DBU)PbBr_3_ microrods (MRs) via pressure engineering. The high emission of MRs with a photoluminescence quantum yield (PLQY) of 86.6% under 5.0 GPa was confirmed to originate from the suppression of non-radiative compounding caused by increased organic–inorganic interactions. Through structural analysis and related calculations, it corroborated that the distance between the nearest Br and N atoms, together with the space angles between the neighboring 2 Br–N pairs, was the major factor on the organic–inorganic interaction strength. Furthermore, we found that the alteration in the main compression direction led by the isostructural phase transition at 5.5 GPa caused a contrasting trend in the evolutions of the interaction strength and optical properties. This study would fill the gap in the mechanism of organic and inorganic interaction in non-hydrogen-bonded hybrid metal halides, providing novel strategy for designing materials with superior performance.

## Results and Discussion

### Basic characterizations and in situ high-pressure optical experiments

**Fig. 1.**
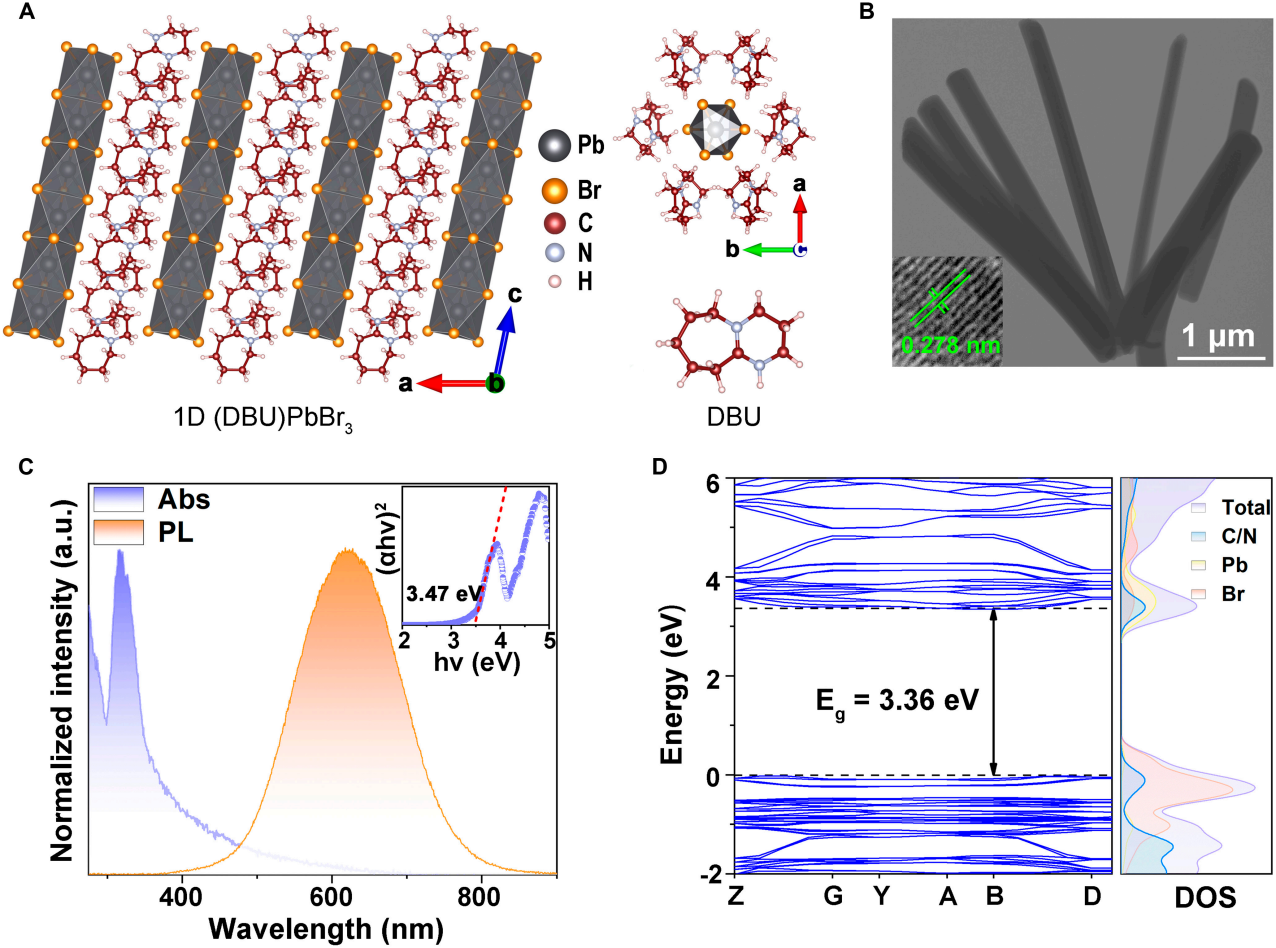
Basic characterization and calculation of (DBU)PbBr_3_ under ambient conditions. (A) Crystal structure of 1D (DBU)PbBr_3_ separately along the [0 1 0] direction (a) and the [0 0 1] direction (c) and the molecular structure of DBU. (B) TEM image of (DBU)PbBr_3_ MRs; inset shows the HRTEM image. (C) Absorption and PL spectra of (DBU)PbBr_3_ MRs and Tauc plot inset. (D) Calculated electronic band structure of (DBU)PbBr_3_. Total and project density of states projected onto the orbitals of C/N, Pb, and Br atoms.

(DBU)PbBr_3_ has a monoclinic crystal structure in the space group of *P*21/*c*, and the crystal structures separately perpendicular to the *b* axis and the *c* axis are displayed in Fig. 1A. The octahedral chains of [PbBr_3_^2+^]_∞_ were separated by the organic cations, [DBU]^+^, leading to a robust quantum confinement effect and a 1D morphology. (DBU)PbBr_3_ exhibited a unique hexagonal arrangement in the direction perpendicular to the *c* axis. Transmission electron microscopy (TEM) images demonstrated that the synthesized sample could be recognized by the micrometer-sized rods with hexagonal cross-sections, which was similar to the arrangement of the microstructure (Fig. 1B). The rods were measured approximately 4.5 μm in length and 400 nm in width, resulting in an aspect ratio above 10. As shown in the absorption spectra, the band edge of MRs was at approximately 357 nm, with a corresponding bandgap of 3.47 eV fitted via the Tauc plot method (Fig. 1C). Under ambient conditions, (DBU)PbBr_3_ MRs exhibited an orange-red emission that covered a wide range of 450 to 800 nm, with a 622-nm central wavelength. The full width at half maximum (FWHM) and Stokes shift reached as large as 189 and 265 nm, respectively. Such a large FWHM width and Stokes shift were clear characteristics of the STE exciton emission [39–41].

First-principles calculations indicated that the bandgap of (DBU)PbBr_3_ MRs was approximately 3.36 eV (Fig. 1D), slightly less than the experimental value, which was caused by the acknowledged periodic boundary embedding (PBE) functional errors. The partial density of states (PDOS) indicated that the valence band of the material was primarily composed of the 4p orbitals of Br and the 2p orbitals of C/N, while the conduction band was primarily composed of the 6p orbitals of Pb, the 4p orbitals of Br, and the 2p orbitals of C/N. The involvement of the organic component DBU in the energy band highlighted the presence of connections between the organic and inorganic components within the (DBU)PbBr_3_ MRs.

**Fig. 2.**
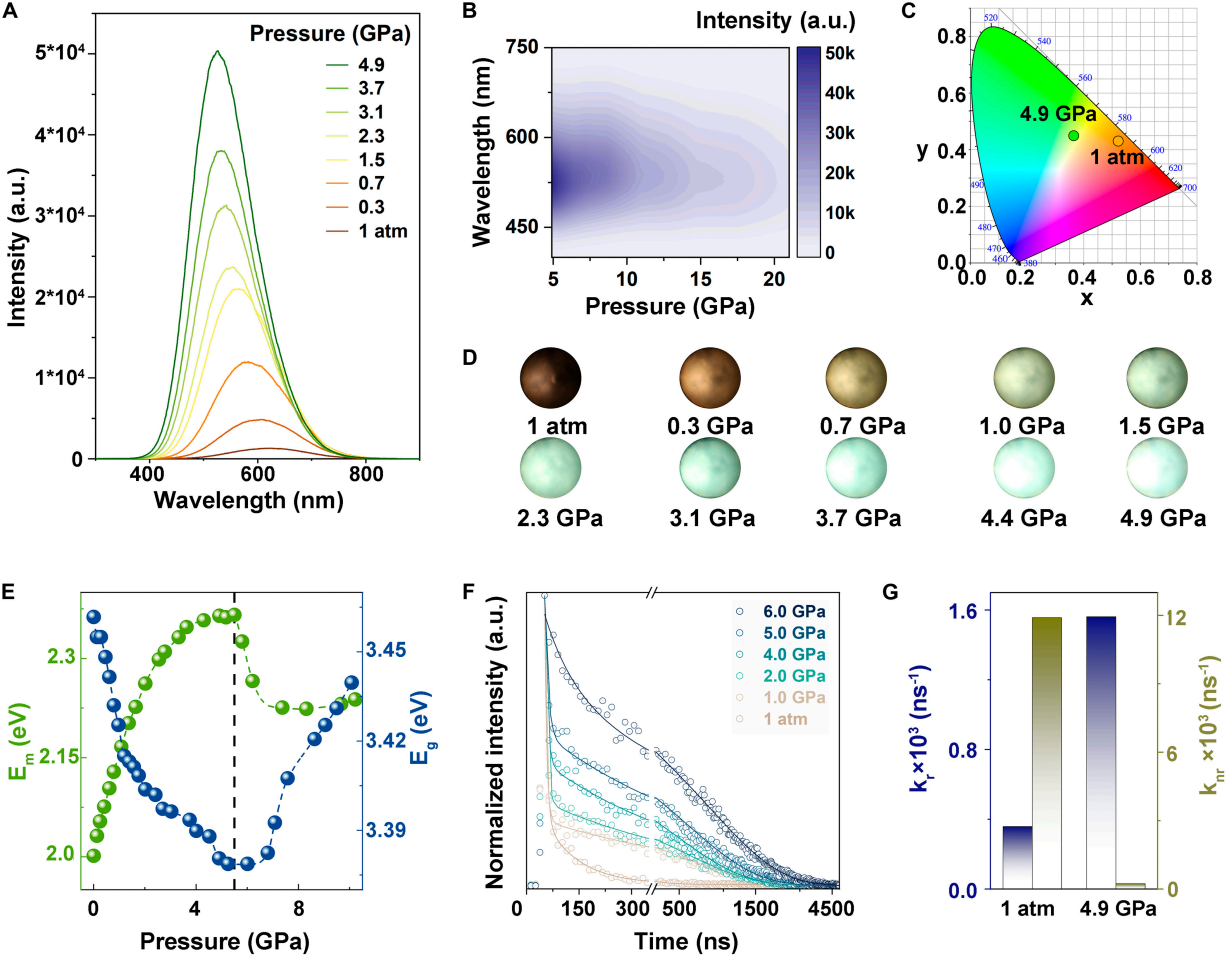
Optical characterization of (DBU)PbBr_3_ under pressure. (A) High-pressure PL evolution of (DBU)PbBr_3_ MRs upon compression to 4.9 GPa. (B) 2D hypsographic map of PL spectra of (DBU)PbBr_3_ MRs within 4.9 to 21.0 GPa. (C) Chromaticity coordinate diagram at 1 atm and 4.9 GPa. (D) PL micrographs upon compression under 355-nm photoexcitation. (E) Emission position and bandgap changes with increasing pressure. (F) TRPL decay curves of (DBU)PbBr_3_ MRs upon compression to 6.0 GPa. (G) Comparison of radiation recombination rate k_r_ (blue) and non-radiative recombination rate k_nr_ (green) at 1 atm and 4.9 GPa.

In order to explore the origin of the organic–inorganic interplay, we conducted high-pressure study on (DBU)PbBr_3_ MRs. Pressure engineering on optical properties included the in situ high-pressure PL, ultraviolet–visible (UV–vis) absorption spectra, and the time-resolved PL (TRPL) experiments. It was found that the PL of (DBU)PbBr_3_ MRs displayed an intense stress response, with an obvious enhancement and blue-shift occurring (Fig. 2A). At a pressure of 4.9 GPa, the emission intensity increased by over 30 times together with a 96-nm shift of peak position as compared to those produced under normal conditions. As a result, the color of emission transformed from dark orange to bright green with pressure (Fig. 2C and D). Subsequently, the PL intensity exhibited a gradual decrease with additional compression, and the peak experienced a later redshift at 5.5 GPa (Fig. 2B). In contrast to the PL spectra, the absorption spectra of MRs displayed minimal changes under pressure, which shifted within 10 nm at the initial pressure (Fig. 2E and Fig. S2). When pressure reached 11.1 GPa, a notable shift toward red of the absorption spectra was observed. This could be attributed to the increased overlap of atomic orbitals under pressure, which led to a significant reduction in the bandgap. In addition, it is noteworthy that absorption and PL of (DBU)PbBr_3_ MRs exhibited contrasting peak shift trends within the same range of pressure. We further calculated the PLQY of MRs at various pressures, and the relevant calculated parameters were shown in Table S1 [42,43]. The (DBU)PbBr_3_ MRs exhibited a high emission of over 86% PLQY at 4.9 GPa, almost 30 times greater than that under ambient conditions (2.9%; Fig. S4).

The TRPL decay curves under pressure were collected to gain insights into carrier dynamics and investigate the underlying factors influencing the PLQY of MRs. As shown in Fig. 2F and Fig. S3, the normalized PL intensity decay slowed down with time dramatically with increasing pressure and subsequently accelerated gradually after 6.0 GPa. There was probably a connection between the intensified emission and the reduced decay rate. By using a biexponential fit, we calculated the average PL lifetime, the radiative recombination rate k_r_, and the non-radiative recombination rate k_nr_ (Supplementary Materials). The τ_i_ and α_i_, listed in Table S2 denoted the lifetime and proportion of each component, respectively. The average PL lifetime rose from 81.5 ns under ambient conditions to about 800 ns under 6.0 GPa and then decreased. Upon analysis of the variations in τ and α of the 2 components, it was found that the noteworthy increase in average PL lifetime was a result of an extension of lifetime and a reduction in the proportion of the short-lived component (Fig. S5). The short-lived lifetime reached 87.2 ns at 6.0 GPa, suggesting that the PL process of the MRs remained unaffected by short-lived processes at that time. Furthermore, k_r_ rose from the initial 0.0036 to 0.012 under 4.9 GPa, while k_nr_ underwent a significant decrease (Fig. 2G and Fig. S6). This suggested that the pressure-induced emission enhancement in the (DBU)PbBr_3_ MRs originated from suppression of the non-radiative composite component by pressure.

### High-pressure structural characterizations

**Fig. 3.**
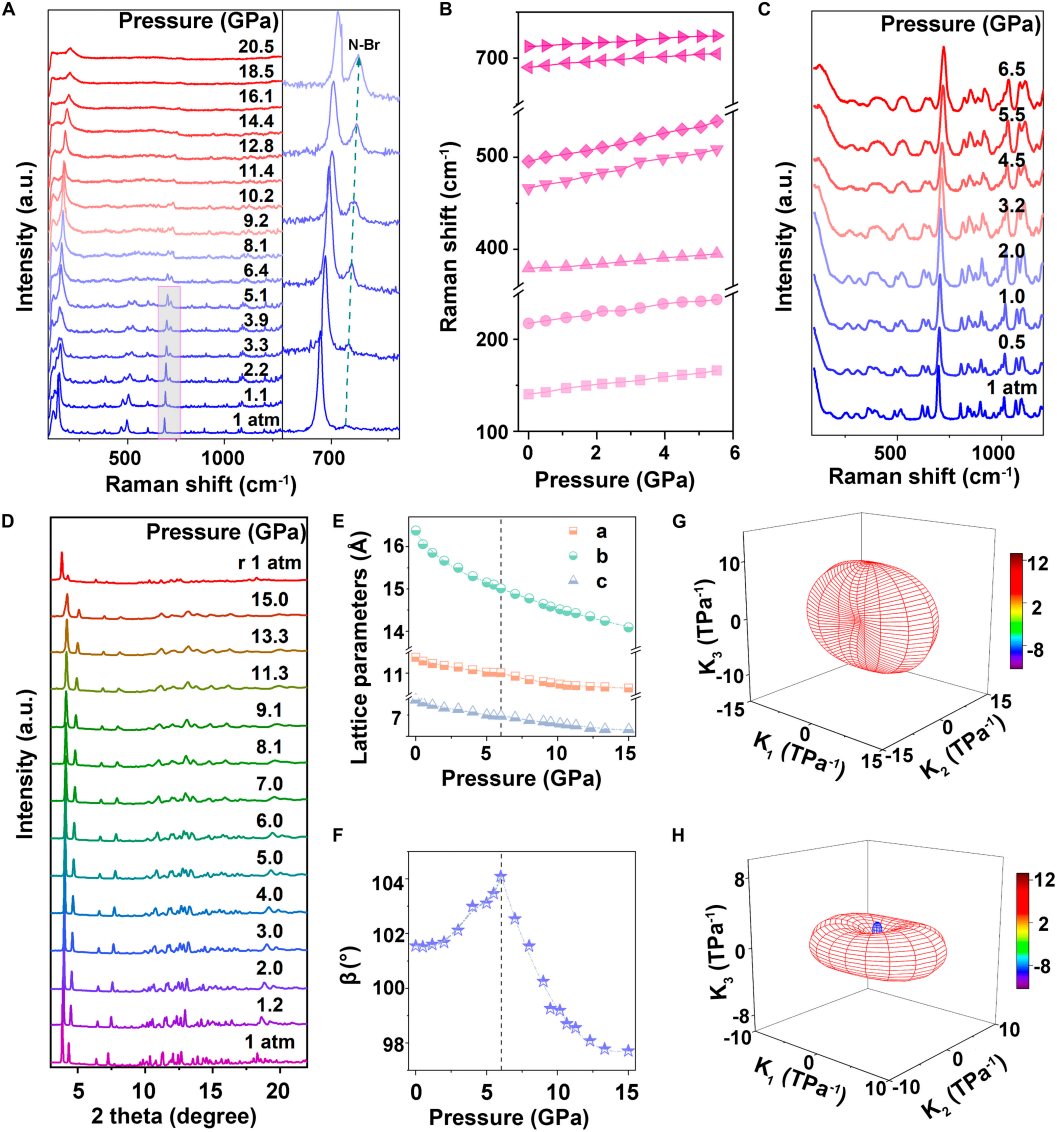
Pressure-induced structure evolution. (A) High-pressure Raman spectra of (DBU)PbBr_3_ MRs upon compression to 20.5 GPa. Localized zoom within 5.1 GPa around 700 cm^−1^. (B) Pressure-dependent evolution of peak positions of some major diffraction peaks. (C) High-pressure Raman spectra of DBU upon compression to 6.5 GPa. (D) High-pressure XRD patterns of (DBU)PbBr_3_ MRs upon compression to 15.0 GPa and decompressed. (E and F) Pressure-dependent evolution of lattice parameters (E), and the angle between the *a*-axis and *c*-axis β (F). (G and H) Evolution of compressibility indicatrix under high pressure below 5.5 GPa (G) and from 5.5 GPa to 15.0 GPa (H). Orthogonal principal axes *X*_1_, *X*_2_, and *X*_3_ are determined based on the eigenvectors of the full strain tensor.

We conducted high-pressure Raman experiments and monitored the evolution of the vibration modes with pressure to establish the correlation between the structure and optical properties of MRs. As shown in Fig. 3A, the Raman intensity of the majority of the vibration modes shifted toward higher wavenumbers and weakened under increasing pressure. When the pressure reached 14.4 GPa, the basic Raman peaks disappeared, indicating that the MRs changed toward amorphization. Note that the vibration mode at approximately 710 cm^−1^ displayed unusual pressure-induced enhancement below 5.1 GPa and subsequently diminished upon further compression. It is found that the enhancement range of the Raman peak aligned with that of the pressure-induced emission enhancement of MRs, which suggested a potential connection between them. Initially, we thought that the high wavenumber vibration mode might have resulted from the high-order harmonic or sum frequency process of fundamental vibration modes, and pressure has facilitated the superposition of these modes additionally. However, when comparing the peak shift of that enhanced mode with the peak shifts of other vibrational modes under pressure (Fig. 3B), we found that the particular one shifted more slowly than the other vibration mode. This implied that the vibration was more probable to be a fundamental vibration mode rather than a combination of other modes. In addition, we also carried out high-pressure Raman experiments on the organic compound, DBU, as a comparison. As shown in Fig. 3C, the high-pressure Raman spectra of DBU did not exhibit abnormally enhancement at comparable vibrational frequencies. Considering that the vibrations of Pb–Br bonds within the inorganic framework match up with low wavenumbers, the enhanced mode did not originate from either the organic or inorganic component exclusively, but rather from the interaction between the 2 components and corresponding to the vibration mode at 710 cm^−1^. The initially weak peak suggested that the interaction was not robust under ambient conditions; however, as the pressure increased, the strength of the interaction was enhanced within 5.1 GPa. The vibration modes of bonds between Br and N were well established at approximately 700 cm^−1^ [44], which corresponded to our findings. As previously mentioned in the energy band analysis of the (DBU)PbBr_3_ MRs, the orbitals of C and N in the organic component were involved in the composition of energy bands. Therefore, the impact of pressure on the optical properties of MRs should stem from its influence on the interaction between organic and inorganic components.

**Fig. 4.**
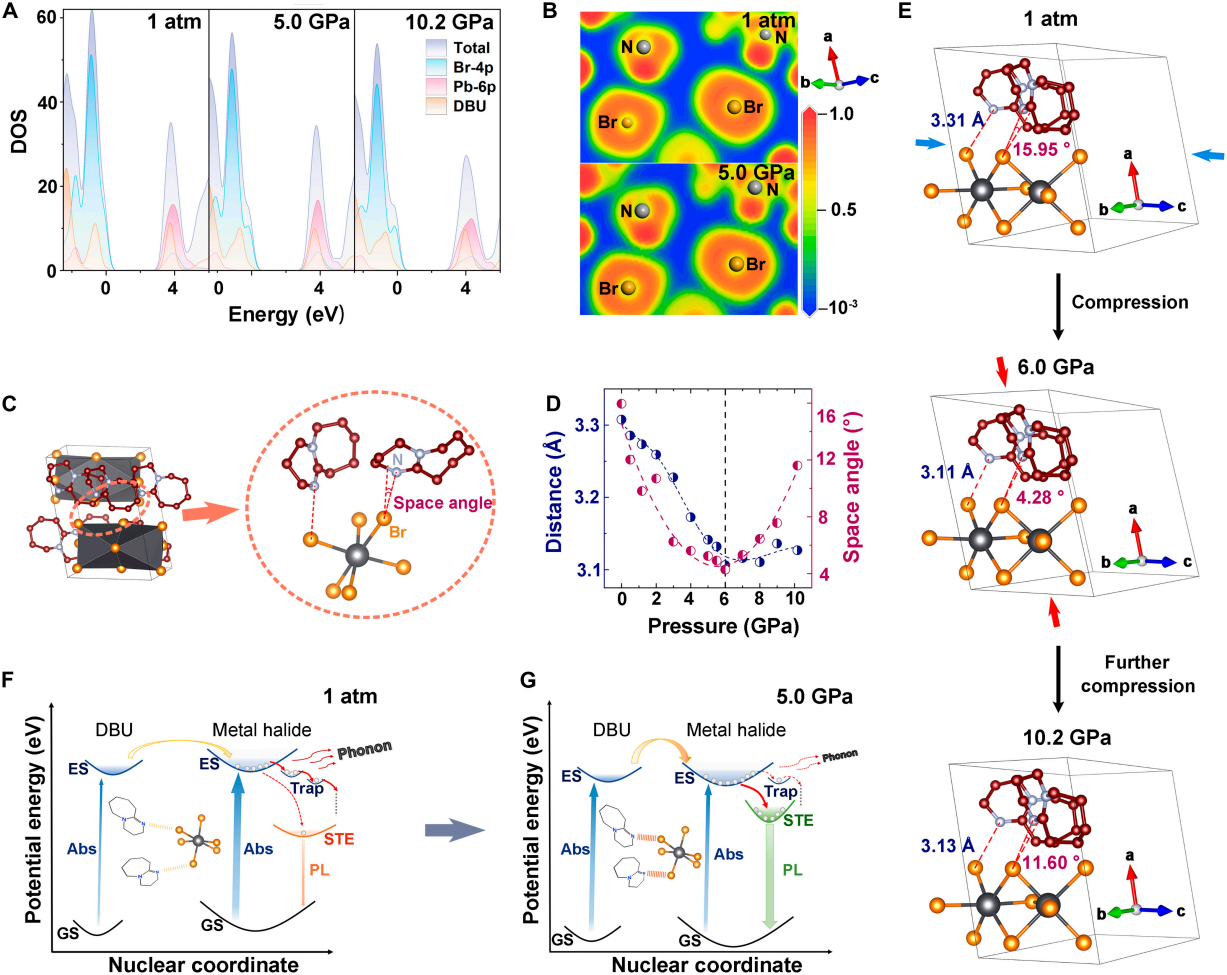
Basic characterization and calculation of (DBU)PbBr_3_ under ambient conditions. (A) Calculated total and partial density of states projected onto the orbitals of DBU, Pb-4p, and Br-4p at 1 atm, 5.0 GPa, and 10.2 GPa, respectively. (B) TEM image of (DBU)PbBr_3_ MRs; inset shows the HRTEM image. (C) ELF map of (DBU)PbBr_3_ MRs. (D) Distance of Br_3_–N_2_ pairs and space angle of the 2 pairs under high pressure. (E) Schematic diagram of Br_3_–N_2_ pairs under different compression direction within the range of 1 atm to 6.0 GPa and 6.0 GPa to 10.2 GPa. (F and G) Schematic diagram of pressure-dependent interaction and emission mechanism at (F) 1 atm and (G) 5.0 GPa. GS, ground state; ES, excited state; STE, self-trapped excitons state.

High-pressure synchrotron angle-dispersive x-ray diffraction (ADXRD) was carried out to investigate the origin of the organic–inorganic interaction through the evolution of structure under high pressure (Fig. 4D). Apparently, no structural phase transition occurred within the pressure range of up to 15 GPa, apart from the Bragg diffraction peaks shifting toward higher angles. The Rietveld refined lattice parameters of MRs under different pressures are shown in Fig. 3E. All 3 axes *a*, *b*, and *c* decreased with increasing pressure, while the β angle exhibited an initial increase until the pressure reached 5.5 GPa, and subsequently decreased (Fig. 3E and F). Further analyzing the pressure-dependent compressibility, MRs showed an anisotropic structural contraction below 5.5 GPa, followed by a slight negative compressibility at the direction of *X*_3_ = 0.56*a* + 0. 83*c* from 5.5 GPa to 15.0 GPa. This suggested that there was an isostructural phase transition occurring at 5.5 GPa, leading to a change in the main direction of compression of the material. This pressure point was equivalent to the inflection pressure point for the evolution of optical properties.

### Related calculation and structure analysis

In order to further explore alterations in optical properties under pressure in relation to the organic–inorganic internal interaction of the (DBU)PbBr_3_ MRs, we calculated the pressure-dependent band structure, PDOS, and electron localization function (ELF). The bandgap of MRs at 5.0 GPa was calculated to be 3.22 eV (Fig. S9), which was consistent with the slight red-shifted absorption spectra under pressure. It was found that the composition of the conduction band remained essentially unchanged with pressure, while the organic component’s proportion in the valence band showed a significant increase from 0 to 5.0 GPa (Fig. 4A and Figs. S10 and S11). This implied that organic components played an increasingly significant role in the photophysical processes of MRs under high pressure. The ELF plots’ comparisons revealed a differentiation in the Br–N (Br_3_–N_2_) pairs, indicating a significant enhancement in their interaction under 5.0 GPa (Fig. 4B). The interatomic spacing between the 2 atoms was found to decrease from 3.31 Å under ambient conditions to 3.11 Å at 6.0 GPa (Fig. 4D). In addition, it was noteworthy that the distance began to increase slightly upon further compression, attributing to the negative compressibility in a specific direction caused by an isostructural phase transition. The modulation of Br–N distances by pressure eventually affected the strength of organic–inorganic interactions within the material. Accordingly, the nearest Br_3_–N_2_ pair of Br and N atoms was identified as the interaction sites between the organic and inorganic components.

Upon examination of the 2 Br_3_–N_2_ atom pairs, the space angle between them tended to decrease first and then to increase (Fig. 4C and D). In general, the interaction between vibrations in space is the result of a superposition of vectors. This leads to the parallel parts adding and the perpendicular parts cancelling out, which means that the decreasing space angle will also make the interactions’ superposition at the macroscopic level more apparent. The variation of the space angle together with the change of the interatomic distances thus enhanced the interplay between the organic and inorganic elements under pressure up to ~6.0 GPa. This also caused the anomalous enhancement of the Raman peak at about 710 cm^−1^ under high pressure. The variable interplay subsequently affected the photophysical properties of the (DBU)PbBr_3_ MRs under pressure. Greater interaction strength increased the overlap of the orbitals, leading to a decrease in the bandgap and an enhanced charge transfer conducive to fluorescence. At the same time, enhanced interactions may reduce the probability of excited state particles being captured by trap states, as evidenced by a significant reduction in the non-radiation recombination rate. The emission of the sample was further enhanced by the suppressed non-radiative recombination. In addition, excited particles were closer to the organic component during the photophysical process, which resulted in a reduced electron–phonon coupling effect and a pronounced blue shift of the emission peaks.

## Conclusion

In summary, the organic–inorganic interaction sites and influence on the enhanced cyan emission in non-hydrogen-bonded 1D hybrid metal halide (DBU)PbBr_3_ were unraveled by means of high pressure. Through structural analysis and first-principles calculations, we confirmed that the decreased distance between the nearest Br and N atoms, together with the decreased space angles between 2 Br_3_–N_2_ pairs, was the major factor on the organic–inorganic interaction strength. Furthermore, the inhibited non-radiative compounding and enhanced charge transfer, owing to pressure-tuned interactions, result in a significantly high PLQY of 86.6%, approximately 30 times higher than the original value. The enhanced cyan emission is particularly important for underwater communication due to the much less attenuation in water than at other wavelength emissions. Likewise, we observed that altering the primary compression direction at 5.5 GPa resulting from the isostructural phase transition gave rise to a negative compressibility in specific direction. This has additional effects on a contrasting trend in the pressure response of the organic–inorganic interactions. Our study elucidates the underlying photophysical mechanism in non-hydrogen-bonded hybrid metal halides, thus providing worthwhile guidelines for materials design with targeted optical performance.

## Materials and Methods

### Synthesis

Lead bromide (PbBr_2_, 99.99%), 1,8-diazabicyclo [5.4.0]undec-7-ene (DBU, 99%), octadecene (ODE,90%), oleyl amine (OLA, 70%), and n-hexane (99%) were purchased from Aladdin, and oleic acid (OA, 90%) was purchased from Sigma-Aldrich. All regents were used directly without further purification.

In the typical synthesis, first, 36.7 mg (0.1 mmol) of lead bromide, 8 ml of ODE, 1 ml of OA, and 1 ml of OLA were placed in a 50-ml 3-necked flask, and the air in the flask was evacuated by passing nitrogen to isolate the effect of aqueous oxygen. The mixture was stirred and heated up to 120 °C until complete clarification of solution was achieved, and subsequently cooled down to 70 °C and stabilized for half an hour. After that, 50 μl of DBU liquid was directly injected directly into the mixture, and the reaction was carried out for 30 s and quenched using an ice water bath. The mixture was centrifuged in some centrifuge tubes at 4,000 rpm for 5 min, and the precipitate was collected and then re-dispersed into n-hexane to mix well. Subsequently, the mixture was repeated centrifuging at 4,000 rpm again for 3 times and the final precipitate was dried to obtain the sample. (DBU)PbBr_3_ MRs were in the form of a white powder.

### Morphology characterization

TEM and high-resolution TEM (HRTEM) images were observed by JEM-2200FS with an emission gun operating at 200 kV. Scanning electron microscopy image was observed by a JSM-6700F, Japan.

### First-principles calculations

All calculations were performed by first-principles density functional theory (DFT), as implemented in the CASTEP package code. Geometry optimizations were calculated using the plane-wave pseudopotential method with the generalized gradient approximation (GGA) based on DFT with CASTEP package. The starting structure was obtained from the Cambridge Structure Database. Here, the electron–ion interactions were described by the projected augmented-wave pseudopotentials, with the C 2s_2_2p_2_, N 2s_2_2p_3_, Pb 6s_2_6p_2_, and Br 4s_2_4p_5_ electrons treated explicitly as valence electrons. The kinetic energy cutoff of 360 eV and the Brillouin zone sampling for geometry optimizations were performed using a 3 × 3 × 5 Monkhorst–Pack *k*-point mesh. The convergence thresholds were set at values of 1.0 e^−5^ eV/atom for energy and 0.03 eV Å^−1^ for force.

### High-pressure generation

High-pressure experiments were performed using a symmetric diamond anvil cell (DAC) equipped with 400-μm anvil diamonds. A T301 steel gasket was pre-indented to a thickness of 45 μm before a 150-μm-diameter hole was drilled in the center. The gasket was then reset to ensure that the indentation fits the anvil face and fixed on one side of the DAC unit. The space between the hole on the gasket and the anvil surface was used as the sample chamber. Pressure was ascertained through the ruby fluorescence technique. A suitable size ruby was placed in the sample chamber using a fine needle, and its fluorescence peak position under ambient pressure (excited by a 405-nm laser) was measured for calibration. Subsequently, the powdery (DBU)PbBr_3_ MRs were picked into the sample chamber and some silicon oil was dropped filling the sample chamber as the pressure-transmitting medium (PTM) with surrounding the sample. Finally, the 2 sides of the DAC were merged to make the sample chamber sealed. In order to ensure to eliminate the influence of pressure hysteresis effect, the test and pressure calibration were carried out after holding for several minutes each pressure point during the experiment.

### In situ high-pressure optical experiments

A semiconductor laser with an excitation wavelength of 355 nm was employed for all fluorescence experiments. The laser beam controlled by an adjustable attenuator was focused on the sample with a 20-μm spot 20× UV Plan apochromatic objective and passed through a filter to eliminate effects on acquisition. Note that all the parameters are fixed completely over each high-pressure PL experiment to avoid the effects of different excitation laser intensities and luminous fluxes on the resulting PL intensity of intrinsic (DBU)PbBr_3_ MRs. Absorption spectra were measured in the exciton absorption band region using a Deuterium–Halogen light source. High-pressure absorption and PL spectra were recorded with an optical fiber spectrometer (Ocean Optics, QE65000). Two seconds was used as the integral time for PL spectra experiments, while 500 ms was used for absorption spectra experiments. Raman spectra were recorded by a Raman spectrometer (SP2500i, Acton, Princeton Instruments) with a 532-nm and 10-mW excitation laser, and the integral time was 10 s. The samples were photoexcited by a 378-nm picosecond pulsed diode laser (PicoHarp, LDH-D-C-405M). The resultant PL was collected and coupled into a grating spectrometer (Princeton Instruments, SP-2558), which directed the spectrally dispersed PL onto an intensified charge-coupled device (ICCD, PI-MAX4, Princeton Instruments) or a photo-multiplier tube (PMT) connected with a photon-counting detector (PDM series, Micro Photon Devices), whose timing was controlled with a PicoHarp300 TCSPC event timer. The measured PL decay curves were fitted using double exponential functions.

### In situ high-pressure x-ray measurements

The in situ high-pressure angle-dispersive XRD experiments were carried out at the 4W2 High Pressure Station in Beijing Synchrotron Radiation Facility. A focused monochromatic x-ray beam with about 5 μm in diameter and wavelengths of 0.6199 Å was used for the diffraction experiments. CeO_2_ was used as the standard sample to do the calibration. A Mar-345 CCD detector was used to collect the Bragg diffraction rings with an average acquisition time of 90 s per point and then integrated into a 1D profile using the Fit2D program.

## Data Availability

Additional data supporting this work are provided in the Supplementary Materials. Other relevant data can be obtained from the authors under reasonable request.

## Supplementary Materials

Supplementary 1 Data analyses Figs. S1 to S12 Tables S1 to S4 References
