# Supplementary material for: Identifying Organic–Inorganic Interaction Sites Toward Emission Enhancement in Non-Hydrogen-Bonded Hybrid Perovskite via Pressure Engineering
Source: Research (Wash D C). 2024 Sep 16;7:0476. doi: 10.34133/research.0476 (PMC11403357; doi:10.34133/research.0476)
Supplement: Supplementary 1 — Data analyses Figs. S1 to S12 Tables S1 to S4 References [file research.0476.f1.docx]

Supporting Information

**Identifying Organic-Inorganic Interaction Sites Toward Emission Enhancement in Non-Hydrogen-Bonded Hybrid Perovskite via Pressure Engineering**

Ming Cong, Dianlong Zhao, Jiayi Yang, Guanjun Xiao^*^, and Bo Zou

State Key Laboratory of Superhard Materials, College of Physics, Jilin University, Changchun 130012, China.

^*^Address correspondence to: [xguanjun@jlu.edu.cn](mailto:xguanjun@jlu.edu.cn)

**Data analyses**

The compressibility coefficients ($K_{i}$) were determined using the PASCal program. The equation is

$K_{i}= -\frac{1}{l}\left( \frac{\partial l}{\partial p} \right)_{T}$ (1)

in which $l$ represents the length of the lattice axis, $p$ represents the pressure [1].

The Reflex module combined in Materials Studio was applied for Rietveld refinement. The pressure-volume data were fitted by the third-order Birch-Murnaghan equation of state as follows:

$P=\frac{3}{2}B_{0}\times\left[ \left( \frac{V}{V_{0}} \right)^{-\frac{7}{3}}-\left( \frac{V}{V_{0}} \right)^{-\frac{5}{3}} \right]\times\left\{ 1+\frac{3}{4}({B'}_{0}-4)\times\left[ \left( \frac{V}{V_{0}} \right)^{-\frac{2}{3}}-1 \right] \right\}$ (2)

where $V_{0}$ is the zero-pressure volume, $B_{0}$ is the bulk modulus at ambient pressure, and ${B'}_{0}$ is a parameter for the pressure derivative [2]. All high-pressure experiments were performed at room temperature.

The time resolved PL decay curves were fitted by the double exponential function [3]:

$I\left( t \right)=I_{0}+A_{1}\times\exp\left( -\frac{t}{\tau_{1}} \right)+A_{2}\times\exp\left( -\frac{t}{\tau_{2}} \right)$ (3)

$\tau_{ave}=\frac{\Sigma\alpha_{i}\times{\tau_{i}}^{2}}{\Sigma\alpha_{i}\times\tau_{i}}$,$i=1,2$ (4)

The analyses of recombinatio rates $k_{r}$ and $k_{nr}$ were calculated by the following equation:

$\tau_{ave}=\frac{1}{k_{r}+k_{nr}}$ (5)

$PLQY=\frac{k_{r}}{k_{r}+k_{nr}}$ (6)


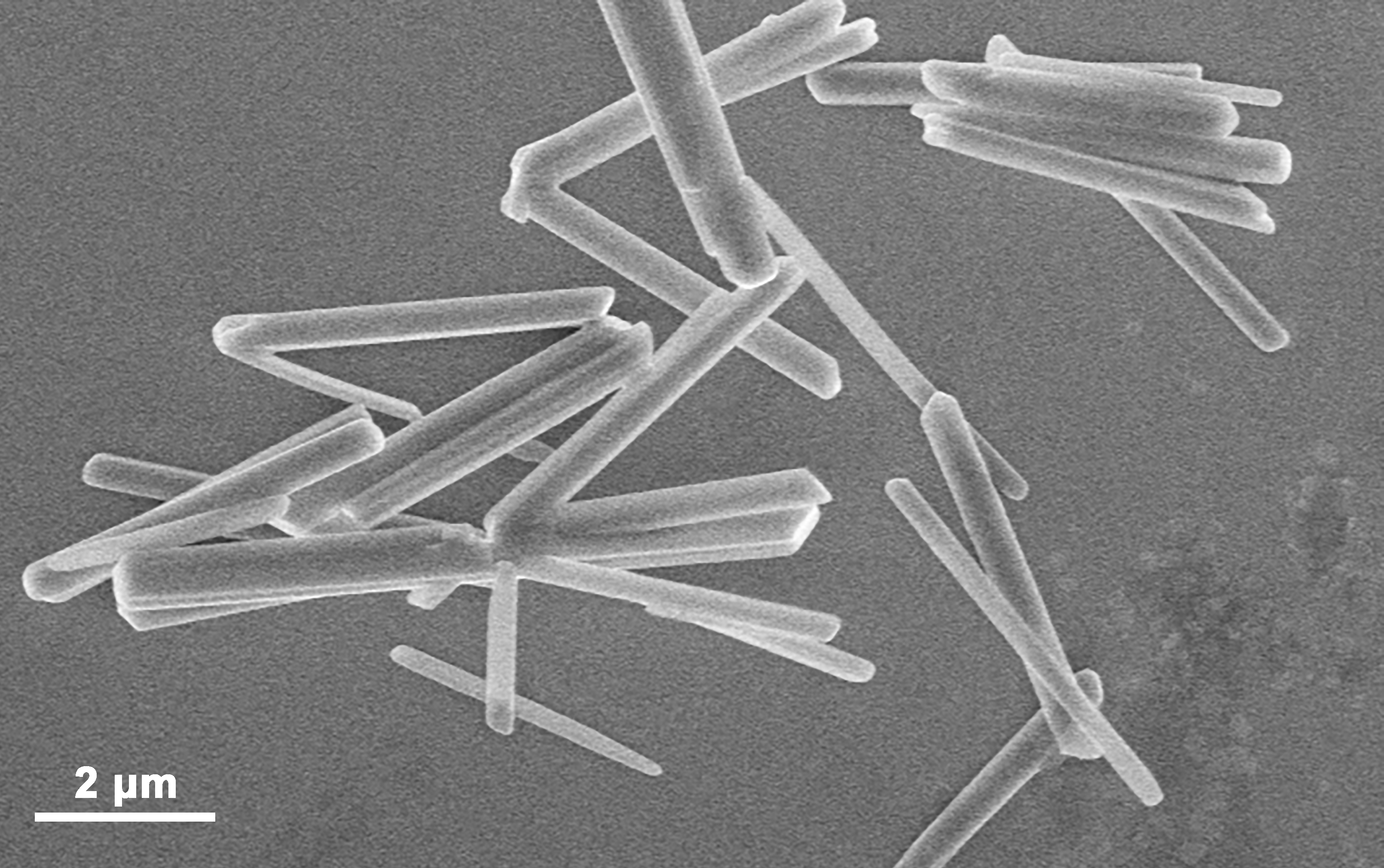


**Fig. S1.** SEM image of (DBU)PbBr_3_ MRs.


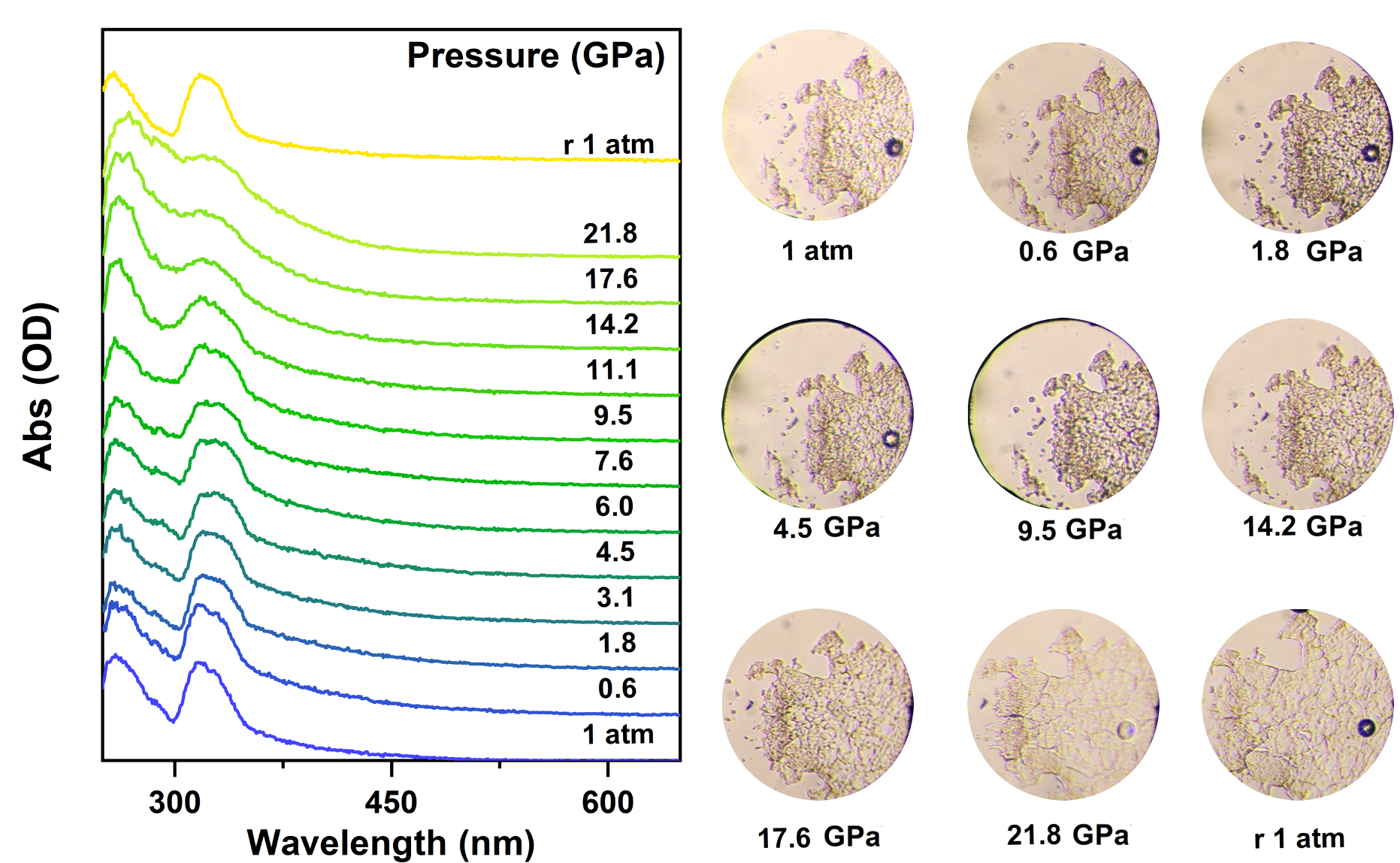


**Fig. S2.** High-pressure abs evolution of (DBU)PbBr_3_ MRs upon compression to 21.8 GPa, and corresponding micrographs.





**Fig. S3.** Time-resolved PL decay curves of (DBU)PbBr_3_ MRs from 6.0 GPa to 12.0 GPa.





**Fig. S4.** Revised PLQY changes with increasing pressure.





**Fig. S5.** Average lifetime a), and proportion of the short-lived lifetime b) changes with increasing pressure.





**Fig. S6.** Radiation recombination rate $k_{r}$ (red) and non-radiative recombination rate $k_{nr}$ (blue) changes with increasing pressure.


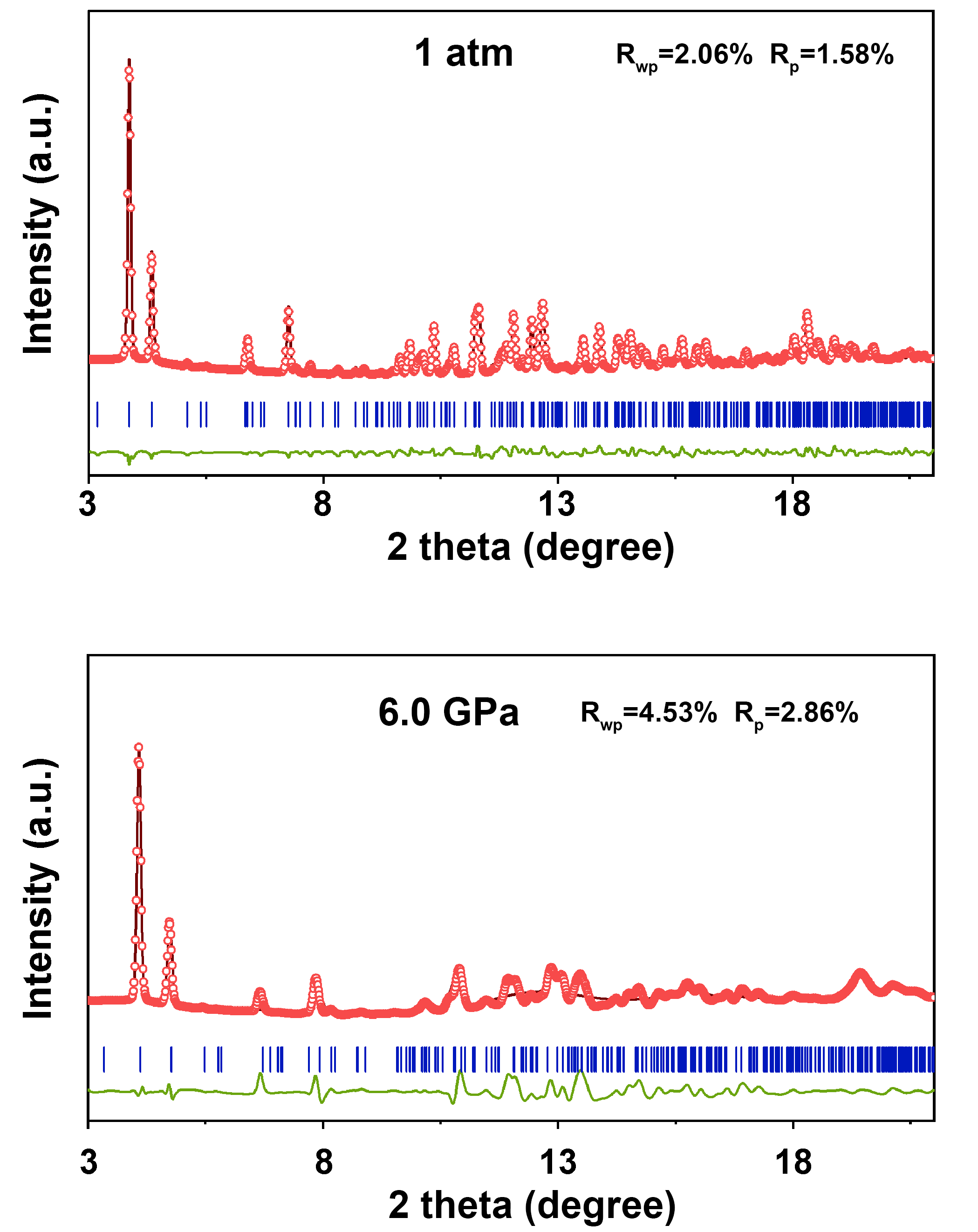


**Fig. S7.** Rietveld refinements at (up)1 atm and (down) 6.0 GPa.


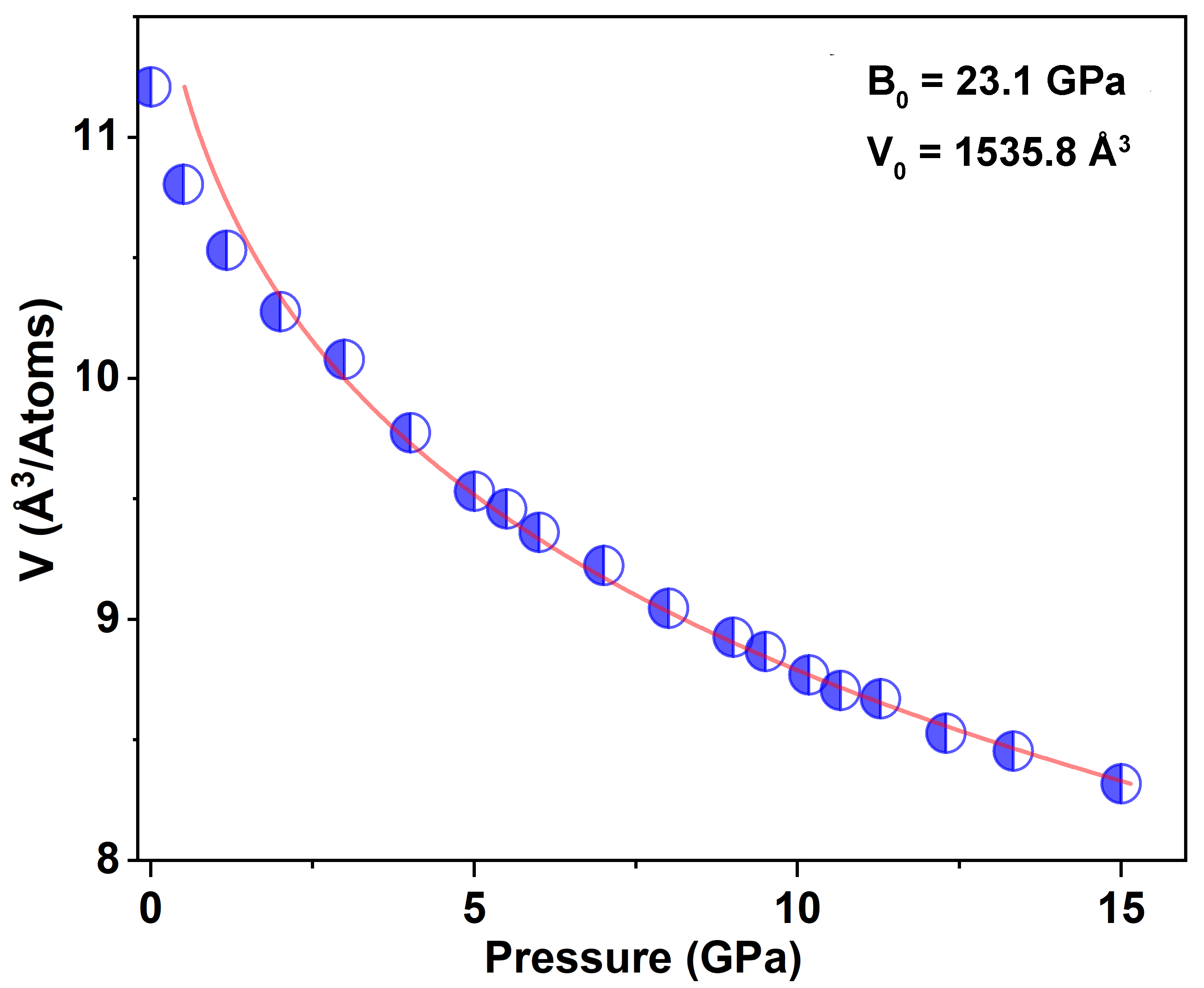


**Fig. S8.** Pressure-dependent evolution of lattice cell volume.





**Fig. S9.** Calculated electronic band structure of (DBU)PbBr_3_ at 5.0 GPa.


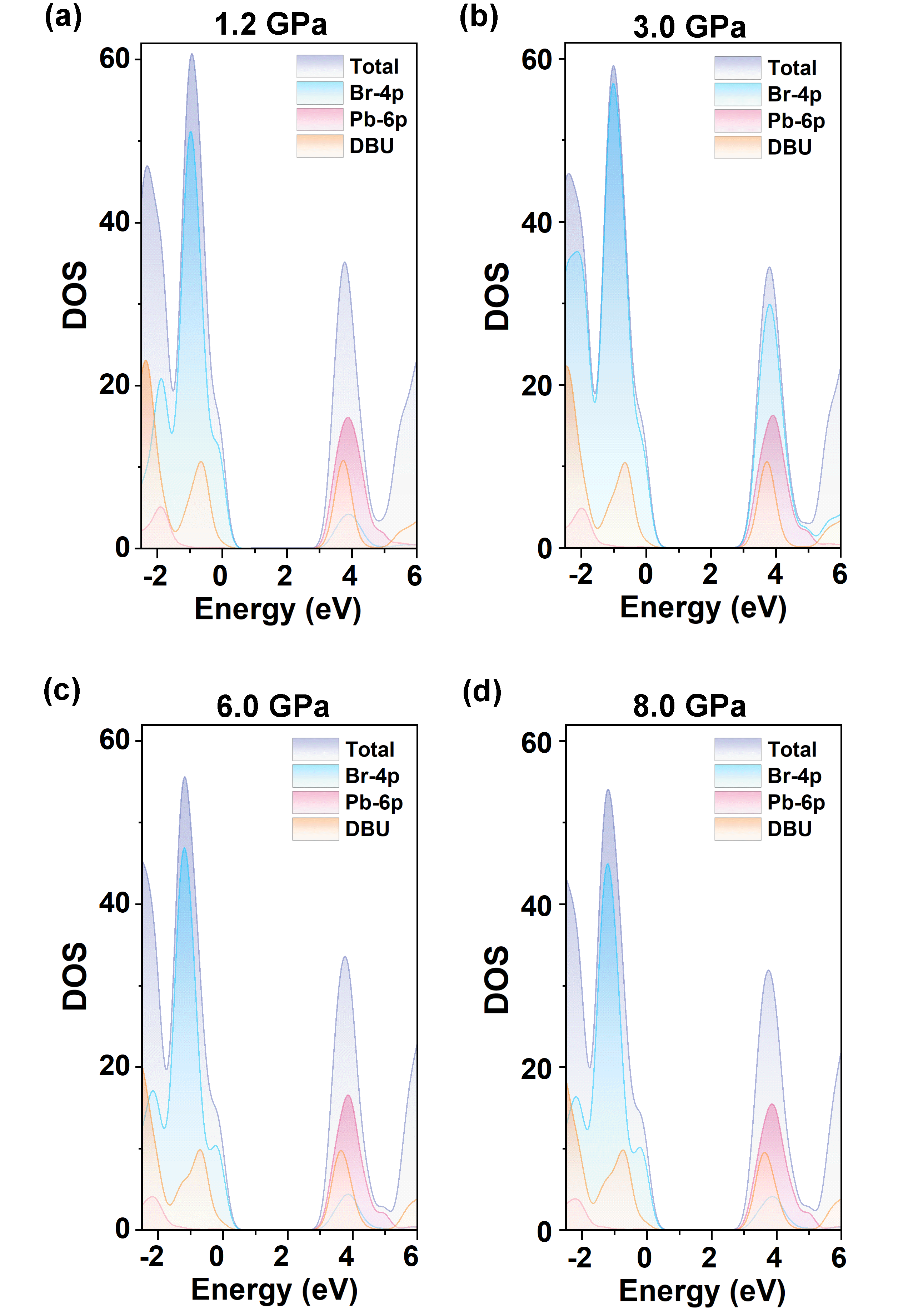


**Fig. S10.** Calculated total and partial density of states projected onto the orbitals of DBU, Pb-4p, and Br-4p at 1.2GPa a), 3.0 GPa b), 6.0GPa c), and 8.0 GPa d) respectively.





**Fig. S11.** Evolution of the proportion of the organic component's proportion in the valence band upon pressure.


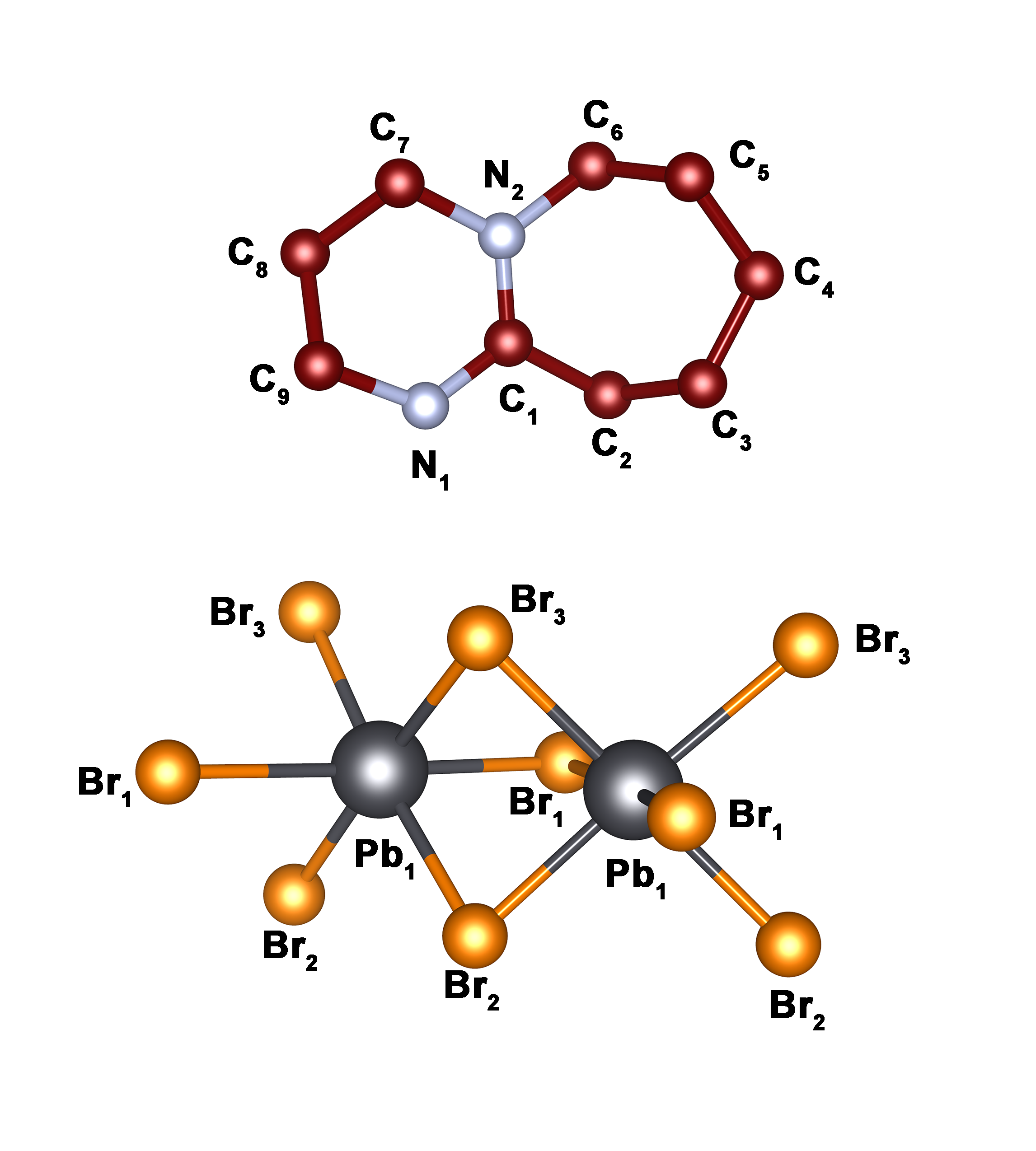


**Fig. S12.** Atomic numbers’ diagram of [DBU]^+^ (up) and the [PbBr_3_^2+^]_∞_octahedral chains (down).

**Table S1.** Revised PLQY under pressure and relevant calculated parameters

| Pressure (GPa) | Integrated intensity | Abs (OD) | ɑ | V | V/V_0_ | n_2_ | PLQY (%) |
| --- | --- | --- | --- | --- | --- | --- | --- |
| 0 | 224142 | 0.14816 | 0.28905 | 1434.79 | 1 | 3 | 2.92702 |
| 0.5 | 1.28E+06 | 0.16809 | 0.32094 | 1383.1 | 1.03737 | 3.12776 | 15.5982 |
| 1.2 | 3.08E+06 | 0.17769 | 0.33578 | 1348.06 | 1.06434 | 3.22407 | 36.83984 |
| 2.1 | 3.45E+06 | 0.19378 | 0.35995 | 1315.52 | 1.09066 | 3.32165 | 39.50206 |
| 3.1 | 4.64E+06 | 0.20282 | 0.37313 | 1290.13 | 1.11213 | 3.40396 | 52.16252 |
| 4.0 | 5.73E+06 | 0.2031 | 0.37353 | 1251.25 | 1.14669 | 3.54195 | 66.40229 |
| 5.0 | 7.30E+06 | 0.2034 | 0.37396 | 1220.19 | 1.17587 | 3.66411 | 86.62891 |
| 5.5 | 6.61E+06 | 0.192 | 0.35731 | 1210.78 | 1.18501 | 3.70348 | 82.79579 |
| 6.3 | 5.75E+06 | 0.18832 | 0.35184 | 1198.32 | 1.19733 | 3.75743 | 73.91686 |
| 7.2 | 5.40E+06 | 0.18625 | 0.34875 | 1180.74 | 1.21516 | 3.83731 | 71.08296 |
| 8.1 | 5.13E+06 | 0.18474 | 0.34648 | 1158.05 | 1.23897 | 3.94752 | 67.62002 |
| 9.0 | 4.31E+06 | 0.17822 | 0.33659 | 1142.66 | 1.25566 | 4.02728 | 57.85858 |
| 9.7 | 4.01E+06 | 0.17391 | 0.32998 | 1135.08 | 1.26404 | 4.06817 | 50.70989 |
| 10.2 | 3.44E+06 | 0.18692 | 0.34975 | 1122.69 | 1.27799 | 4.13744 | 47.38614 |
| 11.0 | 3.17E+06 | 0.212 | 0.38624 | 1114.45 | 1.28744 | 4.18527 | 41.16412 |
| 11.3 | 2.94E+06 | 0.2246 | 0.40379 | 1110.03 | 1.29257 | 4.21153 | 37.0657 |
| 12.8 | 2.65E+06 | 0.2172 | 0.39354 | 1091.73 | 1.31424 | 4.32503 | 33.42971 |
| 13.3 | 2.40E+06 | 0.2246 | 0.40379 | 1082.2 | 1.32581 | 4.38737 | 29.74917 |
| 15.0 | 1.89E+06 | 0.21674 | 0.3929 | 1064.88 | 1.34737 | 4.50687 | 24.43717 |

**Table S2.** Fitting result of the time-resolved PL decay curves of (DBU)PbBr_3_ at different pressure.

| Pressure (GPa) | a_1_ | τ_1_ (ns) | a_2_ | τ_2_ (ns) | τ_ave_ (ns) |
| --- | --- | --- | --- | --- | --- |
| 1 atm | 774.68 | 6.45 | 219.68 | 98.79 | 81.5086 |
| 1.0 | 774.63 | 5.64 | 222.26 | 567.08 | 548.27072 |
| 2.0 | 673.66 | 17.15 | 217.57 | 632.55 | 584.88935 |
| 4.0 | 573.62 | 7.32 | 423.43 | 471.14 | 461.57891 |
| 5.0 | 475.5 | 9.87 | 519.49 | 563.27 | 554.5342 |
| 6.0 | 271.02 | 87.21 | 663.98 | 800.05 | 769.6844 |
| 8.0 | 246.55 | 84.66 | 673.51 | 761.82 | 735.34959 |
| 10.0 | 371.23 | 110.79 | 560.51 | 689.57 | 633.90536 |
| 12.0 | 414.9 | 36.98 | 546.31 | 446.98 | 422.7417 |

**Table S3.** Refinement cell parameters at different pressure.

| Pressure (GPa) | a (Å) | b (Å) | c (Å) | α (°) | β (°) | γ (°) | V (Å^3^) |
| --- | --- | --- | --- | --- | --- | --- | --- |
| 0 | 11.3743 | 16.3801 | 7.86008 | 90 | 101.546 | 90 | 1434.79 |
| 0.5 | 11.2847 | 16.0545 | 7.79122 | 90 | 101.519 | 90 | 1383.1 |
| 1.2 | 11.2245 | 15.851 | 7.73486 | 90 | 101.603 | 90 | 1348.06 |
| 2 | 11.182 | 15.6676 | 7.66758 | 90 | 101.677 | 90 | 1315.52 |
| 3 | 11.1445 | 15.4998 | 7.63918 | 90 | 102.126 | 90 | 1290.13 |
| 4 | 11.0795 | 15.3019 | 7.57429 | 90 | 102.993 | 90 | 1251.25 |
| 5 | 11.0216 | 15.1534 | 7.50147 | 90 | 103.112 | 90 | 1220.19 |
| 5.5 | 11.0178 | 15.0999 | 7.48322 | 90 | 103.458 | 90 | 1210.78 |
| 6 | 11.0063 | 15.0136 | 7.47682 | 90 | 104.092 | 90 | 1198.32 |
| 7 | 10.92775 | 14.8803 | 7.43874 | 90 | 102.536 | 90 | 1180.74 |
| 8 | 10.8417 | 14.7747 | 7.37878 | 90 | 101.543 | 90 | 1158.05 |
| 9 | 10.7983 | 14.6422 | 7.34418 | 90 | 100.25 | 90 | 1142.66 |
| 9.5 | 10.769 | 14.5812 | 7.32405 | 90 | 99.2582 | 90 | 1135.08 |
| 10.2 | 10.7396 | 14.5161 | 7.29511 | 90 | 99.1903 | 90 | 1122.69 |
| 10.7 | 10.712 | 14.4768 | 7.27029 | 90 | 98.7054 | 90 | 1114.45 |
| 11.3 | 10.7026 | 14.4278 | 7.25033 | 90 | 98.5589 | 90 | 1107.1 |
| 12.3 | 10.6857 | 14.3496 | 7.1913 | 90 | 98.0818 | 90 | 1091.73 |
| 13.3 | 10.6738 | 14.2443 | 7.16179 | 90 | 97.7858 | 90 | 1078.85 |
| 15 | 10.6495 | 14.0923 | 7.16036 | 90 | 97.711 | 90 | 1064.88 |

**Table S4.** Refinement cell parameters at different pressure.

|  | Axes | K (TPa^-1^) | Direction | | |
| --- | --- | --- | --- | --- | --- |
|  |  |  | a | b | c |
| Stage Ⅰ | X_1_ | 13.261 | -0.0 | 1.0 | -0.0002 |
|  | X_2_ | 3.2351 | 0.1648 | 0.0 | 0.9863 |
|  | X_3_ | 9.5603 | 0.998 | 0.0 | -0.0631 |
|  | V | 26.3306 |  | | |
| Stage Ⅱ | X_1_ | 8.3521 | -0.5621 | -0.0 | 0.827 |
|  | X_2_ | 6.7756 | 0.0 | 1.0 | -0.0 |
|  | X_3_ | -2.771 | 0.5617 | 0.0 | 0.8273 |
|  | V | 12.695 |  | | |

**References**

[1] Lertkiattrakul M, Evans ML, and Cliffe MJ. PASCal Python: A Principal Axis Strain Calculator. *Journal of Open Source Software.* 2023;8(90):5556. DOI:10.21105/joss.05556

[2] Ma Z, Liu Z, Lu S, Wang L, Feng X, Yang D, Wang K, Xiao G, Zhang L, Redfern SAT, et al. Pressure-Induced Emission of Cesium Lead Halide Perovskite Nanocrystals. *Nature Communications.*2018;9:4506. DOI:10.1038/s41467-018-06840-8

[3] Fišerová E and Kubaka M. Mean Fluorescence Lifetime and Its Error. *Journal of Luminescence.* 2012;32(8):2059-2064. DOI:10.1016/j.jlumin.2012.03.038

[4] Wang Y, Guo S, Luo H, Zhou C, Lin H, Ma X, Hu Q, Du M, Ma B, Yang W, et al. Reaching 90% Photoluminescence Quantum Yield in One-Dimensional Metal Halide C_4_N_2_H_14_PbBr_4_ by Pressure-Suppressed Nonradiative Loss. *Journal of the American Chemical Society.* 2020;142(37):16001-16006. DOI:10.1021/jacs.0c07166

[5] Shi Y, Zhao W, Ma Z, Xiao G, and Bo Z. Self-trapped exciton emission and piezochromism in conventional 3D lead bromide perovskite nanocrystals under high pressure. *Chemical Science.* 2021;12(44):14711-14717. DOI:10.1039/d1sc04987a
